# Development and validation of a prediction model for the incidence of psychological disturbance in Chinese nurses: baseline data from a cohort study

**DOI:** 10.3389/fpubh.2026.1735558

**Published:** 2026-01-27

**Authors:** Xueqian Ma, Zhiqian Chen, Heli Zhang, Rongsong Tang, Hongbo Chen, Baohua Li

**Affiliations:** 1 Department of Radiation Oncology, Peking University Third Hospital, Beijing, China; 2 Department of Geriatrics, Peking University Third Hospital, Beijing, China; 3 Department of Rehabilitative Medicine, Peking University Third Hospital, Beijing, China; 4 Department of Endocrinology, Peking University Third Hospital, Beijing, China; 5 Department of Nursing, Peking University Third Hospital, Beijing, China

**Keywords:** cohort study, nomogram model, nurse, prediction model, psychological disturbance

## Abstract

**Background:**

Healthy psychology is a crucial factor in determining nurses’ ability to provide high-quality nursing care to patients. Therefore, it is essential to detect the risk of nurses’ psychological disturbance and provide early intervention. This study aimed to investigate the psychological status of nurses and develop a nomogram model to predict the incidence of psychological disturbance in Chinese nurses.

**Methods:**

This study was part of the Chinese Nurse Cohort Study, and the data of 3,808 nurses were obtained from multiple tertiary hospitals in China. Data related to psychological disturbance were collected using the Symptom Checklist 90. Predictor selection was guided by the Job Demands-Resources model, encompassing 26 variables across three domains: living conditions, working situation and psychosocial indicators. Predictors were selected via stepwise regression, and a logistic regression model was developed to construct a predictive nomogram. Model performance was evaluated using the area under the receiver operating characteristic curve, decision curve analysis, bootstrap approach and 10-fold cross-validation.

**Results:**

Independent protective indicators for nurses’ psychological disturbance included perceived social support, organizational career management, weekly leisure time, regular meals and published articles, while risk indicators included negative acts, working years, raising children, patients in day shift care and night shift work hours. All these variables were used to establish the nomogram. In the nomogram, the area under the ROC curves was 0.803 (95% *CI*: 0.786–0.819). The average AUC of bootstrap approach was 0.810 (95% *CI*: 0.785–0.817), and the average AUC of 10 fold cross-validation was 0.794 (ranging from 0.749 to 0.841), indicating that the model was stable. The DCA suggested good clinical application.

**Conclusion:**

This study developed a prediction model to evaluate the risk of psychological disturbance among nurses for the first time. Nursing managers can use this visualized prediction model to predict the risk of nurses’ psychological disturbance, identify individualized risk factors, and implement preventive measures to reduce the occurrence of psychological disturbances among nurses.

## 1 Introduction

Nurses in tertiary hospitals faced great work pressure (1). Long-term overwork, high tension, irregular life, complicated interpersonal conflicts, and frequent examinations all put great pressure on nurses’ psychology and affect their physical and psychological health (2). A study with nearly 1,800 nurses from 19 healthcare systems across the United States revealed that more than 50% of the participants reported suboptimal physical and psychological health (3). The psychological disturbance of nurses can lead to a lack of enthusiasm for work, lower work efficiency, and gradual erosion of patience, which can cause a contradiction between nurses and patients and the occurrence of medical errors, leading to poor patient outcomes, lower patient satisfaction and increased costs (4, 5). A previous study indicated that nurses with poor psychological and physical health were 26–71% more likely to report medical errors than those in better health (6). However, while nurses do their best to provide high-quality care to patients, they often do not prioritize their self-care. Severe psychological disturbance can even cause nurse suicide (7). Scholars call for actions to protect the psychological health of medical personnel and to better maintain their long-term health (8); the American Association of Critical Care Nurses, the American Nurses Association, and the Association of Nurse Executives recognize stress in the profession and have called for action to optimize a healthy work environment (9–13); and the National Health Commission of the People’s Republic of China also proposes that various measures be implemented to protect the physical and psychological health of medical staff to build a safer health system (14).

Healthy psychology is a crucial factor in determining nurses’ ability to provide high-quality nursing care to patients. Therefore, it is essential to detect the risk of nurses’ psychological disturbance and provide early intervention. Previous studies investigating the current psychological status of nurses are limited (15, 16), and no tool has been developed to identify the risk of psychological disturbance. Nomograms are statistical models that visually represent complex mathematical formulas and are specifically designed to maximize predictive accuracy (17–19). These models can help determine factors that affect nurses’ psychological health and allow nursing managers to identify at-risk individuals and take preventive measures. Although there have been studies developing prediction models for various health issues in nurses, such as sleep disturbance (20) and fatigue (21), none have focused on predicting the risk of nurses’ psychological disturbance. This study aimed to investigate the psychological status of nurses and develop a nomogram prediction model to assist nurses in maintaining their psychological health.

## 2 Materials and methods

### 2.1 Participants

A convenience sample of registered nurses was recruited from nine tertiary hospitals, and data were collected from March to December 2023. The eligibility criteria for nurses included being registered nurses with full-time employment and providing informed consent to participate in this study. Trainee nurses were excluded. The data were collected via questionnaires through a web-based system. The researchers obtained approval from the principal nurses to allow participants to complete the questionnaires after they explained the purpose of the study to the directors of the nursing departments. Before the questionnaires were distributed, the researchers explained the purpose of the study to prospective participants. The questionnaires were then distributed and collected by the researchers. To adhere to the principle of having at least 10 subjects per variable in the prediction model, a minimum sample size of 250 subjects was required (22). A total of 3,808 nurses were enrolled in this study.

### 2.2 Main outcomes

The incidence of nurses’ psychological disturbance was the main outcome of our study. The Symptom Checklist 90 (SCL-90) was used to collect data related to psychological health. We employed the Chinese version of the SCL-90 translated by Wang (23), which consists of 90 items covering 10 dimensions: somatization, compulsion, interpersonal sensitivity, depression, anxiety, hostility, terror, paranoia, psychosis, and others. Each item is rated on a 5-point Likert scale ranging from 1 (not at all) to 5 (extremely), reflecting the severity of symptoms. The total score, calculated as the sum of all 90 items, ranges from 90 to 450, with higher scores indicating poorer psychological health. Scores for each dimension were computed as the mean of all items within that dimension. A total score exceeding 160, more than 43 positive items (scores ≥ 2), or any dimension score above 2 was considered indicative of a potential psychological disturbance. In this study, the scale demonstrated excellent internal consistency, with a Cronbach’s α coefficient of 0.99.

### 2.3 Predictors and measurement

The selection of potential predictors for the psychological disturbance prediction model was guided by existing literature and framed within the Job Demands-Resources (JD-R) model (24), which highlights the complex and multifactorial nature of nurses’ psychological health. According to the JD-R model and previous studies, psychological outcomes may be influenced by the balance between job demands and available resources. Specifically, job demands-such as workload and shift patterns-may increase stress and contribute to psychological distress (25), while resources like social support and organizational career management probably serve as protective factors that buffer against these adverse effects (26–29). Based on this framework, the predictors included in this study cover three main domains (a total of 26 variables): living conditions (including 9 variables: educational background, average monthly income, marital status, whether to raise children, regular degree of three meals, weekly leisure frequency, weekly leisure time, weekly family companionship frequency, weekly family time), working situation (including 14 variables: hospital list, working years, employment type, working type, daily/night shifts per month, patients in day/night shift care, day/night shift work hours, overtime work hours per week, articles published in recent 5 years, experience of scientific research project, scientific research training experience), and social psychological indicators (including 3 variables: perceived social support, perceived organizational career management, and bullying at work), which were presented in Table 1.

**Table 1:** Baseline characteristics of the total population.

Characteristic	Category	*−x* ± *s/n* (%) (*N* = 3,808)
Perceived social support		66.52 ± 12.00
Organizational career management		54.01 ± 9.09
Negative acts		26.40 ± 7.46
Working years		11.80 ± 8.67
Potential psychological disturbance	No	2,925 (76.8%)
	Yes	883 (23.2%)
Hospital list	Hospital 1	1,936 (50.8%)
	Hospital 2	192 (5.0%)
	Hospital 3	45 (1.2%)
	Hospital 4	274 (7.2%)
	Hospital 5	206 (5.4%)
	Hospital 6	107 (2.8%)
	Hospital 7	8 (0.2%)
	Hospital 8	100 (2.6%)
	Hospital 9	940 (24.7%)
Obtain the highest degree	Technical secondary school	35 (0.9%)
	Junior college	802 (21.1%)
	Undergraduate	2,906 (76.3%)
	Postgraduate and above	65 (1.7%)
Average monthly income	<4,000 RMB	228 (6.0%)
	4,000–8,000 RMB	1,304 (34.2%)
	8,001–12,000 RMB	1,409 (37.0%)
	>12,000 RMB	867 (22.8%)
Marital status	Unmarried	1,219 (32.0%)
	Married	2,471 (64.9%)
	Divorced	98 (2.6%)
	Cohabitation	9 (0.2%)
	Widowed	11 (0.3%)
Whether to raise children	No	1,718 (45.1%)
	Yes	2,090 (54.9%)
Weekly leisure frequency^a^	0 times	297 (7.8%)
	1–3 times	3,334 (87.6%)
	4–6 times	139 (3.7%)
	7–9 times	23 (0.6%)
	> 9 times	15 (0.4%)
Weekly leisure time	<6 h	1,309 (34.4%)
	6–10 h	1,337 (35.1%)
	11–15 h	391 (10.3%)
	16–20 h	278 (7.3%)
	>20 h	493 (12.9%)
Weekly family companionship frequency	<5 times	2,180 (57.2%)
	5–10 times	1,269 (33.3%)
	11–15 times	105 (2.8%)
	>15 times	254 (6.7%)
Weekly family time	<6 h	1,137 (29.9%)
	6–15 h	1,286 (33.8%)
	16–25 h	579 (15.2%)
	26–35 h	284 (7.5%)
	>35 h	522 (13.7%)
Regular degree of three meals	Pretty irregular	188 (4.9%)
	Irregular	510 (13.4%)
	Average	1,768 (46.4%)
	Regular	715 (18.8%)
	Pretty regular	627 (16.5%)
Employment type	Temporary employee	2,263 (59.4%)
	Regular employee	1,545 (40.6%)
Working type	Mental manual labor	579 (15.2%)
	Light manual labor	1,166 (30.6%)
	Moderate manual labor	1,782 (46.8%)
	Heavy manual labor	281 (7.4%)
Daily shifts per month^b^	0	41 (1.1%)
	1–5	414 (10.9%)
	6–10	734 (19.3%)
	11–15	695 (18.3%)
	16–20	616 (16.2%)
	>20	1,308 (34.3%)
Patients in day shift care	<5 patients	860 (22.6%)
	5–8 patients	1,375 (36.1%)
	9–12 patients	724 (19.0%)
	>12 patients	849 (22.3%)
Night shifts per month	0	1,514 (39.8%)
	1–5	602 (15.8%)
	6–10	1,050 (27.6%)
	11–15	591 (15.5%)
	16–20	34 (0.9%)
	>20	17 (0.4%)
Patients in night shift care	0 patients	1,265 (33.2%)
	1–8 patients	804 (21.1%)
	9–16 patients	334 (8.8%)
	17–24 patients	345 (9.1%)
	>24 patients	1,060 (27.8%)
Day shift work hours	<9 h	1,823 (47.9%)
	9–12 h	1,906 (50.1%)
	13–16 h	63 (1.7%)
	>16 h	16 (0.4%)
Night shift work hours	<9 h	2,025 (53.2%)
	9–12 h	1,122 (29.5%)
	13–16 h	542 (14.2%)
	>16 h	119 (3.1%)
Overtime work hours per week	<9 h	3,142 (82.5%)
	9–16 h	544 (14.3%)
	17–24 h	68 (1.8%)
	>24 h	54 (1.4%)
Articles published in recent 5 years	0	2,887 (75.8%)
	1–3	872 (22.9%)
	4–6	37 (1.0%)
	>6	12 (0.3%)
Experience of scientific research project	No	3,210 (84.3%)
	Yes	598 (15.7%)
Scientific research training experience	No	2,192 (57.6%)
	Yes	1,616 (42.4%)

a Leisure refers to travel, shopping, sports, reading, listening to music, etc.

b Generally, 8 h is a shift. RMB, The currency of China. h, hours.

#### 2.3.1 Perceived social support scale

The Chinese version of the perceived social support scale (PSSS) was translated by Jiang (30). This scale measures three dimensions: family support, friend support, and other support. Twelve items were scored based on a 7-point Likert scale ranging from 1 (extremely disagree) to 7 (extremely agree). A higher score reflects better perceived social support. In this study, the Cronbach’s α coefficient of the scale was 0.96.

#### 2.3.2 Organizational career management questionnaire

The organizational career management questionnaire (OCMQ) was developed by Long et al. (31) and combined 16 items related to justice in promotion, providing career information, valuing training, and promotion in career self-development. Each item was scored on a four-point Likert scale from 1 (strongly disagree) to 4 (strongly agree). A higher score reflects better perceived organizational career management. In this study, the Cronbach’s α coefficient of the scale was 0.95.

#### 2.3.3 Negative acts questionnaire-revised

The Chinese version of the negative acts questionnaire-revised (NAQ-R) was translated by Xun et al. (32) and combined 23 items related to person-related negative acts, work-related negative acts, and organizational injustice. Each item was scored based on a 5-point Likert scale ranging from 1 (never) to 5 (daily). A higher score reflects greater perceived bullying at work. In this study, the Cronbach’s α coefficient of the scale was 0.95.

### 2.4 Statistical analysis

Statistical analysis was performed using R software version 4.3.2. Continuous variables were expressed as mean (*x̄*) ± standard deviation (*s*), and categorical variables were expressed as frequencies (*n*) and percentages (%). We selected the most statistically significant predictors using stepwise regression based on the Akaike Information Criterion (AIC) criterion. The model used logistic regression, and the probability of psychological disturbances in nurses was predicted using a binomial distribution. We assessed multicollinearity by calculating the Variance Inflation Factor (VIF) for each variable. Generally, a VIF value less than 5 indicates a low likelihood of multicollinearity, suggesting minimal correlation between the variables and a more stable model. Based on the stepwise regression results, the nomogram model was developed, which was used to predict the probability of nurses’ psychological disturbance and score the proportion of each factor in the model. A receiver operating characteristic (ROC) curve was used to evaluate the discriminatory ability of the prediction model. The greater the area under the ROC curve (AUC), the greater the discrimination of the prediction model. An AUC greater than 0.7 indicates a reasonable estimate (19). To assess the clinical benefit of the prediction model, we used Decision Curve Analysis (DCA). DCA measures the clinical benefit of the model by calculating the standardized net benefit at different thresholds. The net benefit takes into account the costs of false positives and false negatives and visually demonstrates the model’s performance at various risk thresholds.

To further validate the stability of the model, we performed 1,000 bootstrap resamples. In each resample, we randomly selected samples with replacement from the training dataset and evaluated the AUC of the model generated from each resample. AUC was used as the performance metric, and we reported the average AUC along with its 95% confidence interval (*CI*). To assess the generalizability and stability of the model, we applied 10-fold cross-validation. In each fold, the dataset was randomly divided into 10 subsets, with 9 subsets used for training the model and the remaining subset used for testing. The evaluation metrics for cross-validation included AUC, sensitivity, and specificity, and we adjusted the model’s threshold to optimize the balance between sensitivity and specificity. To evaluate whether hospital-level heterogeneity affects the predictive ability of the model, we used a generalized linear mixed-effects model (GLMM), considering hospital as a random effect. There were no missing values in the predictive variables in this study. Two-tailed *p* < 0.05 was considered to indicate statistical significance.

## 3 Results

### 3.1 The general characteristics of nurses

A total of 3,808 nurses were included in our study, 883 (23.2%) of whom were considered to have a potential psychological disturbance. 2,471 (64.9%) nurses were married, and the average working years of nurses was 11.80 ± 8.67. There are 2,906 undergraduate nurses, accounting for 76.3%. Nurses’ income levels were mainly concentrated in 4,000–8,000 RMB (34.2%) and 8,001–12,000 RMB (37.0%). Additional information was shown in Table 1. In addition, the results showed that the factor scores of obsessive compulsive (1.53 ± 0.56), somatization (1.41 ± 0.47), and depression (1.39 ± 0.52) ranked in the top three (Table 2).

**Table 2:** The general psychological health status of nurses.

Dimensions	Factor scores (*N* = 3,808)
Somatization	1.41 ± 0.47
Obsessive compulsive	1.53 ± 0.56
Interpersonal sensitivity	1.29 ± 0.47
Depression	1.39 ± 0.52
Anxiety	1.33 ± 0.47
Hostility	1.34 ± 0.49
Phobic anxiety	1.20 ± 0.39
Paranoid ideation	1.21 ± 0.41
Psychoticism	1.21 ± 0.40

### 3.2 Establishment of the prediction model

**Figure 1:**
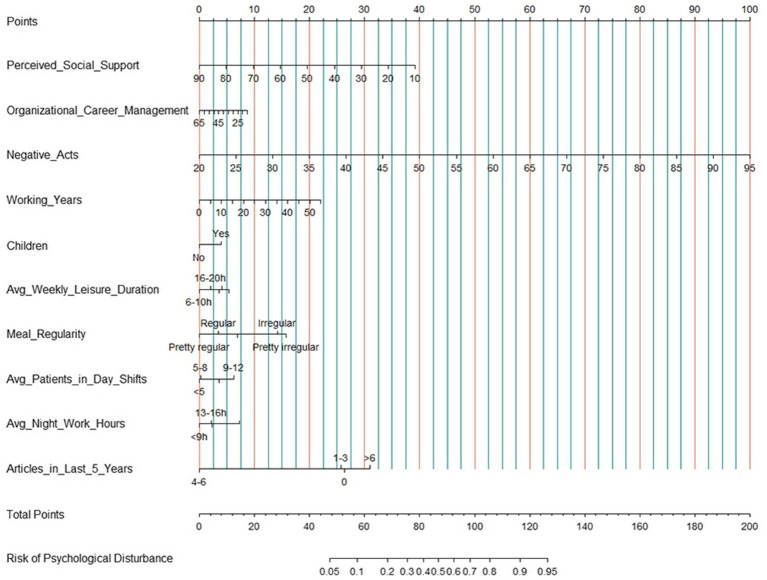
The nomogram prediction model for nurses’ psychological disturbance.

The model was constructed using the entire population sample. The initial model included 26 variables, and 10 significant predictors were ultimately retained through stepwise regression. As shown in Table 3, the independent protective factors included perceived social support, organizational career management, weekly leisure time, meal regularity, and the number of published articles. The independent risk factors included negative acts, working years, raising children, the number of patients in day shift care, and night shift work hours. The VIF for all variables were less than 2, indicating minimal correlation between them. The residual deviance of the model was 3280.5, and the AIC value was 3326.5, suggesting that the model demonstrated a good fit. Based on psychological disturbance as the outcome variable and these 10 independent predictors, a risk prediction model for nurses’ psychological disturbance was developed (Figure 1). The corresponding score for each independent predictor on the score scale at the top of the nomogram model was determined, and the scores of all the predictive factors were summed to calculate the total score. The point where the total scores corresponded to the risk line at the bottom of the nomogram model was the predicted value of the nurses’ psychological disturbance.

**Table 3:** Stepwise regression analysis for risk factors for nurses’ psychological disturbance.

Variable	Category	Estimate	Std. Error	*z* value	*p* value	VIF
Intercept		−0.890	0.470	−1.893	0.058	
Perceived social support		−0.037	0.004	−9.130	<0.001	1.062
Organizational career management		−0.013	0.005	−2.509	0.012	1.092
Negative acts		0.099	0.007	14.769	<0.001	1.075
Working years		0.030	0.006	4.807	<0.001	1.267
Whether to raise children	No	Ref.				1.212
	Yes	0.298	0.109	2.735	0.006	
Weekly leisure time	<6 h	Ref.				1.007
	6–10 h	−0.305	0.107	−2.858	0.004	
	11–15 h	0.096	0.154	0.619	0.536	
	16–20 h	−0.156	0.186	−0.840	0.401	
	>20 h	−0.039	0.143	−0.275	0.784	
Regular degree of three meals	Pretty irregular	Ref.				1.027
	Irregular	−0.107	0.206	−0.522	0.602	
	Average	−0.659	0.191	−3.459	0.001	
	Regular	−0.913	0.215	−4.241	<0.001	
	Pretty regular	−1.169	0.226	−5.165	<0.001	
Patients in day shift care	<5 patients	Ref.				1.022
	5–8 patients	0.018	0.125	0.148	0.883	
	9–12 patients	0.465	0.136	3.421	0.001	
	>12 patients	0.270	0.132	2.047	0.041	
Night shift work hours	<9 h	Ref.				1.035
	9–12 h	0.170	0.108	1.574	0.116	
	13–16 h	0.160	0.134	1.197	0.231	
	>16 h	0.541	0.230	2.350	0.019	
Articles published in recent 5 years	0	Ref.				1.017
	1–3	−0.053	0.105	−0.504	0.614	
	4–6	−1.968	0.648	−3.036	0.002	
	>6	0.337	0.791	0.426	0.670	

VIF, variation inflation factors. Ref., reference group.

### 3.3 Validation of the prediction model

**Figure 2:**
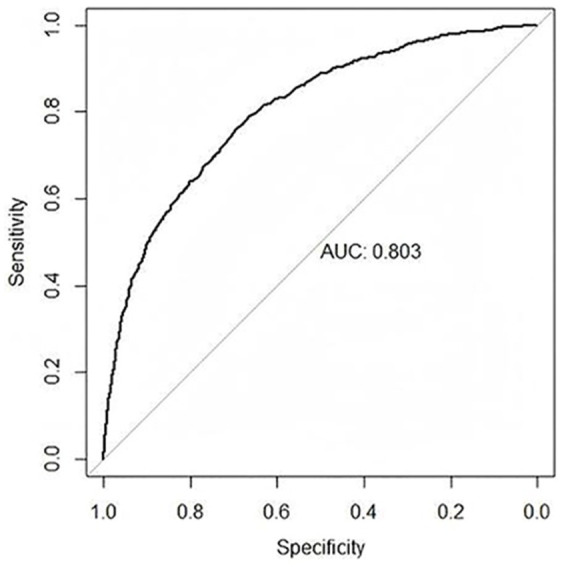
Receiver operating characteristic curves for the nomogram prediction model.

**Figure 3:**
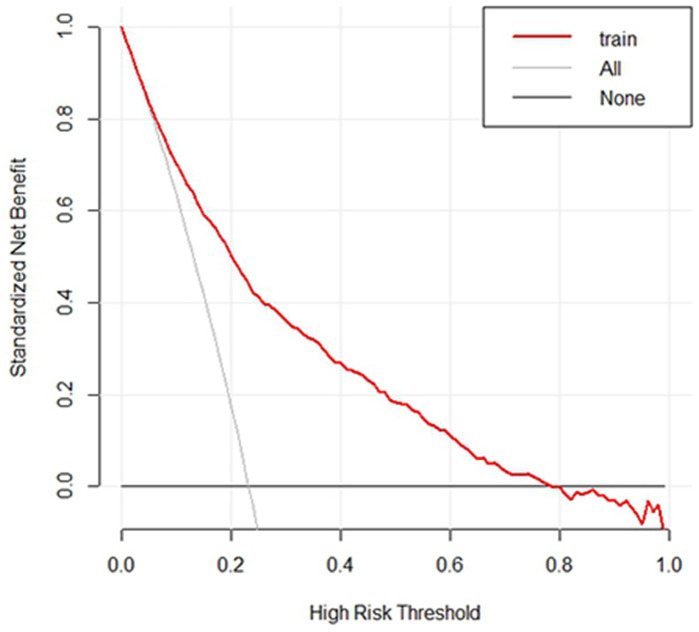
Decision curve analysis for the nomogram prediction model.

The AUC value for the final model was 0.803 (95% *CI*: 0.786–0.819), which showed that the nomogram had good discrimination and prediction abilities (Figure 2). Furthermore, the average AUC value calculated using the bootstrap approach was 0.810 (95% *CI*: 0.785–0.817), with a standard error of 0.0078, indicating that the model demonstrated good stability across different sample sampling conditions. Through 10-fold cross-validation, we assessed the model’s performance. Across all folds, the model’s AUC ranged from 0.749 to 0.841, with an average AUC of 0.794, suggesting that the model exhibited strong classification performance. After adjusting the threshold (from 0.5 to 0.8), the model’s sensitivity was 0.794, and its specificity was 0.784, successfully balancing the impact of false positives and false negatives. This adjustment made the model suitable for classification tasks in practical applications. The DCA results indicated good clinical benefit of the nomogram (Figure 3). The area between the model curve and the black and light gray lines indicated the net benefit of this model in predicting nurses’ psychological disturbance. Compared to the simple strategies of classifying “all nurses at risk” or “no nurses at risk,” the model showed higher clinical benefits across different thresholds, particularly within the range of 0.1 to 0.8, where the net benefit was maximized. It is recommended to select this range for clinical decision-making. Additionally, the GLMM results indicated that the variance of random effects between hospitals was minimal (variance = 0.001, standard deviation = 0.037), indicating that heterogeneity across hospitals had minimal impact on the final model, supporting the decision to pool the data from all nine hospitals.

## 4 Discussion

Based on the baseline data of 3,808 nurses in a set study, we developed and validated the first nomogram prediction model to predict the incidence of psychological disturbance in nurses. The model demonstrated excellent discrimination and clinical value, which indicated that perceived social support, organizational career management, weekly leisure time, regular meals and published articles were protective indicators, while negative acts, working years, raising children, patients in day care and night shift work hours were risk indicators. Identifying the specific challenges they face will allow nursing managers to offer customized interventions.

Among the 3,808 nurses in this study, 883 (23.2%) were considered to have a positive psychological disturbance, a lower rate than that of emergency department nurses (32.9%) (33). This difference may be attributed to the fact that the nurses in this study came from various departments, with those in nonemergency departments experiencing lower work pressure and workload. The factor scores for obsessive-compulsive symptoms were the highest. Chinese nurses are required to strictly adhere to the system ‘three confirmations and eight checks’ (check and confirm patient name, patient’s bed number, drug name, drug dose, drug concentration, route of administration, time of administration and date of expiration of the drug before, during and after nursing operation) to prevent errors in their work, leading to the development of a habit of repeated checks and examinations, resulting in higher scores and positive rates for obsessive-compulsive symptoms (34).

Social support was identified as a key factor in promoting the psychological well-being of nurses. Nursing managers should implement strategies such as effective communication, emotional support, spiritual guidance, tangible rewards, and other forms of support to help nurses manage stress and maintain a positive psychological state (35). The positive relationship between organizational support and mental health has been widely reported, and it theoretically includes specific organizational measures such as career management. Enhancing career growth and meeting basic psychological needs can improve psychological safety and a sense of belonging, thereby reducing the risk of psychological disturbances (36, 37). Nursing managers who support nurses’ career development and work environment can thus foster a healthier psychological status among their workforce by enhancing work-related resources that counterbalance job demands and reduce distress. Leisure activities help in emotional recovery from stressful work, alleviate stress, and reduce anxiety/depressive symptoms. The positive effects of this have been empirically supported in nursing intervention studies (38).

Furthermore, this study suggested that regular consumption of three meals is a protective factor for nurses’ psychological health, similar to the findings of the study by Hossain et al. on university students (39). The impact of dietary habits on psychological well-being may be related to the gut microbiota (GM). Hormones and neurotransmitters produced by the GM can influence behavioral and emotional responses (40). Nursing managers should schedule shifts in a way that ensures that nurses have regular meals. In this study, we also found that nurses who published 4–6 articles in the last 5 years had the lowest risk of psychological disturbance. The number of articles published is closely related to title promotion; too few articles can hinder promotion and career advancement. In contrast, nurses who publish a greater number of papers may face increased research responsibilities. Nursing managers should establish a reasonable requirement for the number of articles published when considering title promotion qualifications to balance the impact of publishing articles on nurses.

This study revealed that nurses with children are more likely to experience psychological disturbances, probably due to the time, energy, and financial resources required for raising children (41). Similar to previous research (42–44), this study showed that nurses with longer tenure are more likely to experience psychological disturbance. As nurses gain more experience, their levels of occupational stress and job burnout tend to increase (45). To address burnout among senior nurses, nurse managers should consider implementing measures such as appropriate salary adjustments and increased annual leave days. Furthermore, the results indicated that nurses who managed more patients during the day shift or who worked longer in the night shift were at a greater risk of experiencing psychological disturbances. Night shifts commonly disrupt circadian rhythms, leading to significant changes in sleep and biological functions that can affect physical and psychological well-being (46). Research suggests that nurses require at least 4 days to adjust their cortisol secretion rhythms after a night shift (47), but this is challenging due to limited nursing resources. Nursing managers should minimize the duration of night shifts whenever possible.

This study also showed that nurses who experienced workplace bullying were more likely to suffer psychological disturbance, consistent with previous research (48). Victims of workplace bullying can experience feelings of depression, humiliation, vulnerability, or threat, leading to increased pressure and decreased self-confidence, ultimately resulting in resignation (49). Nursing managers should implement interventions to reduce workplace bullying and cultivate a professional, supportive work environment.

### 4.1 Implications for nursing management

This study developed a prediction model to evaluate the risk of psychological disturbance among nurses for the first time. The results have great implications for the ability of nursing managers to provide effective psychological support to nurses. Nursing managers can utilize this visualized nomogram prediction model to predict nurses’ risk of psychological disturbance and identify individualized risk factors. The findings can help nursing managers maintain nurses’ psychological health, and they can provide a reference for implementing preventive measures to reduce the occurrence of psychological disturbance among nurses. Nursing managers can implement effective communication, emotional support, spiritual guidance, tangible rewards, and other forms of support to help nurses manage stress and maintain a positive psychological state. By formulating relevant positive policies, nursing managers can reduce occupational stress and workplace bullying, create a professional and supportive working environment, and ultimately improve the quality of nursing work.

### 4.2 Limitations

Limitations exist in this study. First, data from a single follow-up cannot establish a strict causal relationship between variables. To clarify causality, longitudinal data or panel data are needed for further research. Second, the data were based on self-reports, which can lead to reporting bias, as respondents might exaggerate or conceal psychological health symptoms. Future studies should consider cross-referencing self-reports with clinical records and health and social services records.

## 5 Conclusion

A prediction model was developed and validated in this study to predict the incidence of psychological disturbance among nurses. The results indicated that nurses in tertiary hospitals may have a greater likelihood of experiencing obsessive-compulsive psychological states. Protective indicators included perceived social support, organizational career management, weekly leisure time, regular meals and published articles, while risk indicators of psychological disturbance included negative acts, working years, raising children, patients in day care and night shift work hours. The model demonstrated excellent discrimination and clinical value, offering significant implications for identifying and preventing psychological disturbances among nurses for nursing managers and ultimately improving nurses’ psychological well-being.

## Data Availability

The raw data supporting the conclusions of this article will be made available by the authors, without undue reservation.
